# Pharmacokinetics and safety of GST-HG171, a novel 3CL protease inhibitor, in Chinese subjects with impaired and normal liver function

**DOI:** 10.1128/aac.00539-24

**Published:** 2024-07-11

**Authors:** Jing Zhou, Hong Zhang, Hong Chen, George Zhang, John Mao, Tianxiang Zhang, Yanan Tang, Wenhao Yan, Chuanjing Li, Yanhua Ding, Qinglong Jin

**Affiliations:** 1 Phase I Clinical Research Center, The First Hospital of Jilin University, Jilin, China; 2 Fujian Akeylink Biotechnology Co., Ltd., Fuzhou, Fujian, China; 3 Department of Hepatology, The First Hospital of Jilin University, Jilin, China; IrsiCaixa Institut de Recerca de la Sida, Barcelona, Spain

**Keywords:** clinical trial, hepatic impairment, pharmacokinetics, safety, GST-HG171, COVID-19

## Abstract

GST-HG171 is a potent, broad-spectrum, orally bioavailable small-molecule 3C-like (3CL) protease inhibitor that was recently approved for treating mild to moderate coronavirus disease 2019 patients in China. Since cytochrome P450 (CYP) enzymes, primarily CYP3A, are the main metabolic enzymes of GST-HG171, hepatic impairment may affect its pharmacokinetic (PK) profile. Aiming to guide clinical dosing for patients with hepatic impairment, this study, using a non-randomized, open-label, single-dose design, assessed the impact of hepatic impairment on the PK, safety, and tolerability of GST-HG171. Patients with mild and moderate hepatic impairment along with healthy subjects were enrolled (*n* = 8 each), receiving a single oral dose of 150 mg GST-HG171, with concurrent administration of 100 mg ritonavir to sustain CYP3A inhibition before and after GST-HG171 administration (−12, 0, 12, and 24 hours). Compared to subjects with normal hepatic function, the geometric least-squares mean ratios (90% confidence intervals) for GST-HG171’s maximum plasma concentration (*C*_max_), area under the concentration-time curve up to the last quantifiable time (AUC_0-*t*_), and area under the plasma concentration-time curve from time 0 extrapolated to infinity (AUC_0-∞_) in subjects with mild hepatic impairment were 1.14 (0.99, 1.31), 1.07 (0.88, 1.30), and 1.07 (0.88, 1.29), respectively. For moderate hepatic impairment, the ratios were 0.87 (0.70, 1.07), 0.82 (0.61, 1.10), and 0.82 (0.61, 1.10), respectively. Hepatic impairment did not significantly alter GST-HG171’s peak time (*T*_max_) and elimination half-life (*T*_1/2_). GST-HG171 exhibited good safety and tolerability in the study. Taken together, mild to moderate hepatic impairment minimally impacted GST-HG171 exposure, suggesting no need to adjust GST-HG171 dosage for patients with mild to moderate hepatic impairment in the clinic.

**Clinical Trials:**

Registered at ClinicalTrials.gov (NCT06106113).

## INTRODUCTION

Since 2022, various variants of the severe acute respiratory syndrome coronavirus 2 (SARS-CoV-2) (such as Omicron: JN.1, BA.2.86, XBB.1.5, etc.) have exhibited a notable decrease in pathogenicity compared to early strains (e.g., Alpha, Beta, Delta, etc.), while demonstrating significantly enhanced transmissibility and immune evasion capabilities. These variants have rendered the population widely susceptible, with the elderly, particularly those with severe underlying health conditions, exhibiting higher rates of severe illness and mortality post-infection than the general population (1–5). As of March 2024, over 774 million confirmed cases of COVID-19 (coronavirus disease 2019) have been reported with more than 7.0 million deaths attributed to the novel coronavirus infection (6).

Amidst the COVID-19 pandemic, various antiviral drugs have emerged, targeting viral proteins such as RNA-dependent RNA polymerase (RdRp) and main protease (Mpro or 3CLpro), as well as host targets like angiotensin-converting enzyme 2 (ACE2) and immune modulation pathways, including Janus kinase/signal transducer and activator of transcription pathways, and monoclonal antibodies (7–12). Of these, 3CL protease inhibitors have garnered significant attention, with agents like nirmatrelvir-ritonavir demonstrating efficacy against multiple coronaviruses, including variants of the novel coronavirus, thereby preventing severe COVID-19 infections in asymptomatic or mildly symptomatic individuals, thus reducing hospitalization and mortality rates (13–16). However, the viral selective pressure may lead to additional mutations in the protease protein, resulting in decreased effectiveness of nirmatrelvir-ritonavir (17). Moreover, given the widespread infection rates globally, many patients cannot afford antiviral drugs, necessitating the development of safer, more effective, and affordable treatment options.

GST-HG171 is a novel, potent, and selective oral 3CL protease inhibitor. It is more potent and effective than nirmatrelvir in preclinical studies both *in vitro* and *in vivo*. GST-HG171 has broad-spectrum activity against different variants of SARS-CoV-2, including Beta, Delta, Omicron B.1.1.529, Omicron BA.4, BA.5 variants with 5–10-fold higher activity than nirmatrelvir when tested head-to-head in cellular assays. *In vivo*, GST-HG171 demonstrated higher efficacy than nirmatrelvir in reducing the viral load of lung tissue in mice infected with SARS-CoV-2 virus. Furthermore, GST-HG171 has a more preferable lung tissue distribution in rats than nirmatrelvir with a 4–5-fold higher lung/plasma exposure ratio. GST-HG171 has an excellent safety profile in both preclinical studies and Phase I clinical studies in healthy human volunteers (18). GST-HG171 exhibits four times higher exposure in human plasma compared to Paxlovid, indicating its potential to be used either as high-dose monotherapy or in combination with low-dose ritonavir (RTV). RTV, a CYP3A4 inhibitor (19), can increase the exposure [*C*_max_ and AUC (area under the curve)] of GST-HG171 in healthy subjects by up to 3–6 times, enabling sufficient multiples of GST-HG171 trough concentration above its EC50 *in vitro*. Predictions from population PK (pharmacokinetic) models suggest that the clinical efficacy of GST-HG171 at 150 mg in combination with 100 mg ritonavir may be comparable to or better than nirmatrelvir (300 mg)/ritonavir (100 mg) (18). The combination of 150 mg GST-HG171 with 100 mg ritonavir was selected for Phase II/III clinical trials and demonstrated excellent efficacy and safety (20). It is currently approved for marketing by the Chinese National Medical Products Administration for treating mild to moderate COVID-19 patients in China.

Preclinical data suggest that cytochrome P450 (CYP) enzymes, primarily CYP3A, are the main metabolic enzymes of GST-HG171. In hepatocytes and liver microsomes, five metabolites have been identified in addition to GST-HG171, indicating that the liver may be the primary metabolic organ. Since patients expected to receive GST-HG171 treatment may have hepatic impairment, increasing their risk of severe COVID-19 (21), and hepatic impairment may affect the PK profile of GST-HG171, leading to changes in the drug’s safety, tolerability, or efficacy, it is necessary to conduct PK and safety studies of GST-HG171 in subjects with hepatic impairment in comparison to healthy controls. The current study presents the results of PK, safety, and tolerability following a single oral administration of 150 mg GST-HG171 in combination with 100 mg ritonavir in subjects with mild to moderate hepatic impairment compared to healthy controls.

## MATERIALS AND METHODS

### Study design

This study employed a single-center, non-randomized, open-label, parallel-group, single-dose trial design to evaluate the pharmacokinetics and safety of a 150-mg GST-HG171 combined with 100 mg ritonavir oral administration in subjects with mild to moderate hepatic impairment and those with normal hepatic function (ClinicalTrials.gov Identifier: NCT06106113). A total of 24 subjects were enrolled and divided into three groups: mild hepatic impairment (Child-Pugh A), moderate hepatic impairment (Child-Pugh B), and healthy controls with eight subjects in each group. Healthy controls were matched with hepatic impairment subjects based on gender and within ±10 kg of average weight and within ±10 years of average age. Subjects underwent screening assessments within 14 days prior to dosing. Eligible subjects underwent baseline assessments 1 day before the trial and were admitted to the study center on day −1. To ensure CYP3A inhibition before dosing, subjects received a single oral dose of 100 mg ritonavir on the evening of day −1. Ritonavir acts rapidly, significantly, and persistently inhibiting CYP3A4 enzyme activity after administration, with maximum inhibition occurring within the first 48 hours of ritonavir initiation (22). Subjects fasted overnight for at least 10 hours and then received a single oral dose of 150 mg GST-HG171 tablet and 100 mg ritonavir tablet with approximately 240 mL of warm water on an empty stomach on day 1 morning. Following dosing, 100 mg ritonavir was administered orally every 12 hours to maintain CYP3A inhibition. Subjects refrained from water intake within 1 hour before and after oral administration of GST-HG171 and from food intake within 2 hours after dosing. Subjects maintained a seated position for 2 hours after GST-HG171 administration and restricted their activity out of bed. Subjects were discharged on day 3 and underwent telephone follow-up (or communication via text message, etc.) on day 7 (±1 day). Safety assessments were conducted through monitoring adverse events, physical examinations, vital signs, laboratory tests, and 12-lead electrocardiograms.

### Subjects

Eligible Chinese male or female subjects meeting the following criteria were included in the study: aged between 18 and 65 years; body mass index (BMI) ranging from 18 to 32 kg/m^2^; creatinine clearance ≥60 mL/min; willing to practice strict contraception from self-screening to 6 months after the last administration of the study drug; hepatic impairment patients should also meet the following criteria: chronic liver damage caused by primary liver diseases (such as hepatitis B, hepatitis C, autoimmune hepatitis, alcoholic liver disease, etc.); Child-Pugh A or B; stable medication history for at least 14 days before the study drug administration, with no adjustments required during the study period, or non-users; normal hepatic function subjects should be matched with hepatic impairment subjects (age, gender, weight). The main exclusion criteria include the following: use of strong or moderate inducers or inhibitors of CYP3A enzymes, strong inhibitors or inducers of P-glycoprotein (P-gp) within 1 month prior to screening; QTc interval (QTcF) >470 ms for males or >480 ms for females on the screening electrocardiogram; severe infection, trauma, gastrointestinal surgery, or other major surgeries within 4 weeks prior to screening; blood donation or loss ≥200 mL within 3 months prior to screening; pregnant or lactating women, or women of childbearing potential with positive pregnancy tests. Hepatic impairment subjects meeting any of the following exclusion criteria should be excluded: past or current severe systemic diseases other than the primary liver disease; laboratory test results at screening meeting any of the following criteria: (i) alanine aminotransferase (ALT) or aspartate aminotransferase (AST) >10× ULN (Upper Limit of Normal), (ii) absolute neutrophil count (NE#) <0.75 × 10^9^/L, (iii) hemoglobin <60 g/L, and (iv) alpha-fetoprotein >100 ng/mL. Normal hepatic function subjects meeting any of the following exclusion criteria should be excluded: use of any prescription drugs, over-the-counter drugs, herbal supplements, or remedies within 14 days before administration of the study drug; abnormal findings on physical examination, vital signs, laboratory tests, 12-lead electrocardiogram, abdominal ultrasound, or any other examinations judged clinically significant by the investigator; history of liver damage.

### PK analysis

Blood samples for PK analysis were collected at 0 hours before GST-171 administration on day 1 (D1) and at 0.25, 0.5, 0.75, 1, 1.25, 1.5, 2, 3, 4, 6, 8, 12, 24, and 48 hours post-dose. Each collection involved drawing 4 mL of venous blood into vacutainer tubes containing EDTA-K2 anticoagulant. Following collection, the tubes were centrifuged at 1,500 × *g* for 10 minutes at 2–8°C to obtain clear plasma. Bioanalysis of GST-HG171 plasma samples was performed using validated liquid chromatography-tandem mass spectrometry methods with sensitivity and specificity. The validation report of the detection method is retained by Frontage Laboratories (SuZhou) Co., Ltd. The concentration range of the standard curve for GST-HG171 ranged from 10 ng/mL to 10,000 ng/mL with a lower limit of quantitation (LLOQ) of 10 ng/mL. The intra-batch accuracy (percentage bias) of the LLOQ concentration level quality control samples ranged from −14.2% to 4.9% with an inter-batch accuracy of 2.0%. The precision and accuracy of the quality control samples met the acceptance criteria for biological samples. For quality control samples other than the LLOQ concentration, the intra-batch accuracy (percentage bias) ranged from −16.0% to −1.0% with an inter-batch accuracy of −3.8% to −1.6%. The precision and accuracy of the quality control samples met the acceptance criteria for biological samples, demonstrating good reproducibility in the analysis of biological samples.

### Safety assessments

Adverse events, physical examinations, vital signs, laboratory tests, and 12-lead electrocardiograms were conducted. The clinical characteristics, severity, onset time, end time, duration, management measures, and outcomes of adverse events were recorded, and their correlation with the study drug was determined. Adverse event severity was assessed according to the Common Terminology Criteria for Adverse Events version 5.0. Treatment-emergent adverse events (TEAEs) were continually monitored during the 3-day hospitalization at the study center and during a follow-up phone call on day 7 (±1 day) after the initial dose of GST-HG171.

### Statistical analysis

The concentration data of GST-HG171 were analyzed using Phoenix WinNonlin version 8.3. Non-compartmental pharmacokinetic parameters were estimated based on the actual blood sample collection time, and the main pharmacokinetic parameters were calculated. These parameters include the following: AUC_0-∞_, AUC_0-*t*_, *C*_max_, *T*_max_, λz, *T*_1/2_, Vz, and CLz. The *C*_max_, AUC_0-*t*_, and AUC_0-∞_ of GST-HG171 were log-transformed and subjected to analysis of variance using a mixed-effects model with liver function as the effect factor. The least-squares difference and its 90% confidence interval for the main pharmacokinetic parameters in different liver function states were calculated. The geometric mean ratio and its 90% confidence interval for the main pharmacokinetic parameters were calculated after taking the antilogarithm, comparing the differences in pharmacokinetic characteristics between subjects with liver function impairment and those with normal liver function. *T*_max_ was compared between different liver function states using the Wilcoxon rank-sum test. Statistical analysis was performed using SAS version 9.4 software.

## RESULTS

Baseline characteristics of subjects demographic data of the subjects are shown in Table 1. All 24 enrolled subjects completed the entire trial, including medication administration, PK blood sampling, and safety observations. Among them, there were 21 males and 3 females, with 22 subjects being Han nationality and 2 from other ethnic groups. Eight subjects had normal liver function, eight had mild liver impairment, and eight had moderate liver impairment. The subjects in the normal liver function group were matched with those in the mild and moderate liver impairment groups in terms of age, gender, and weight.

**TABLE 1:** Baseline demographics and clinical characteristics^*a*^

Baseline parameter	Mild hepatic impairment (*N* = 8)	Moderate hepatic impairment (*N* = 8)	Normal hepatic function (*N* = 8)
Age, years	45.88 ± 5.22	54.25 ± 6.71	46.38 ± 6.05
Sex, male/female	6/2	8/0	7/1
Weight, kg	67.65 ± 11.52	79.40 ± 11.29	70.20 ± 4.20
BMI, kg/m^2^	23.63 ± 2.88	27.25 ± 3.20	24.88 ± 1.81
Ethnicity, *n* (%)			
Han ethnicity	7 (87.5)	7 (87.5)	8 (100)
Others	1 (12.5)	1 (12.5)	0
Child–Pugh score, *n* (%)			
5–6 (mild)	8 (100.0)		NA
7–9 (moderate)		8 (100.0)	NA

^*a*^ Values are mean ± standard deviation or numbers of subjects (%).

### PKs

**Fig 1:**
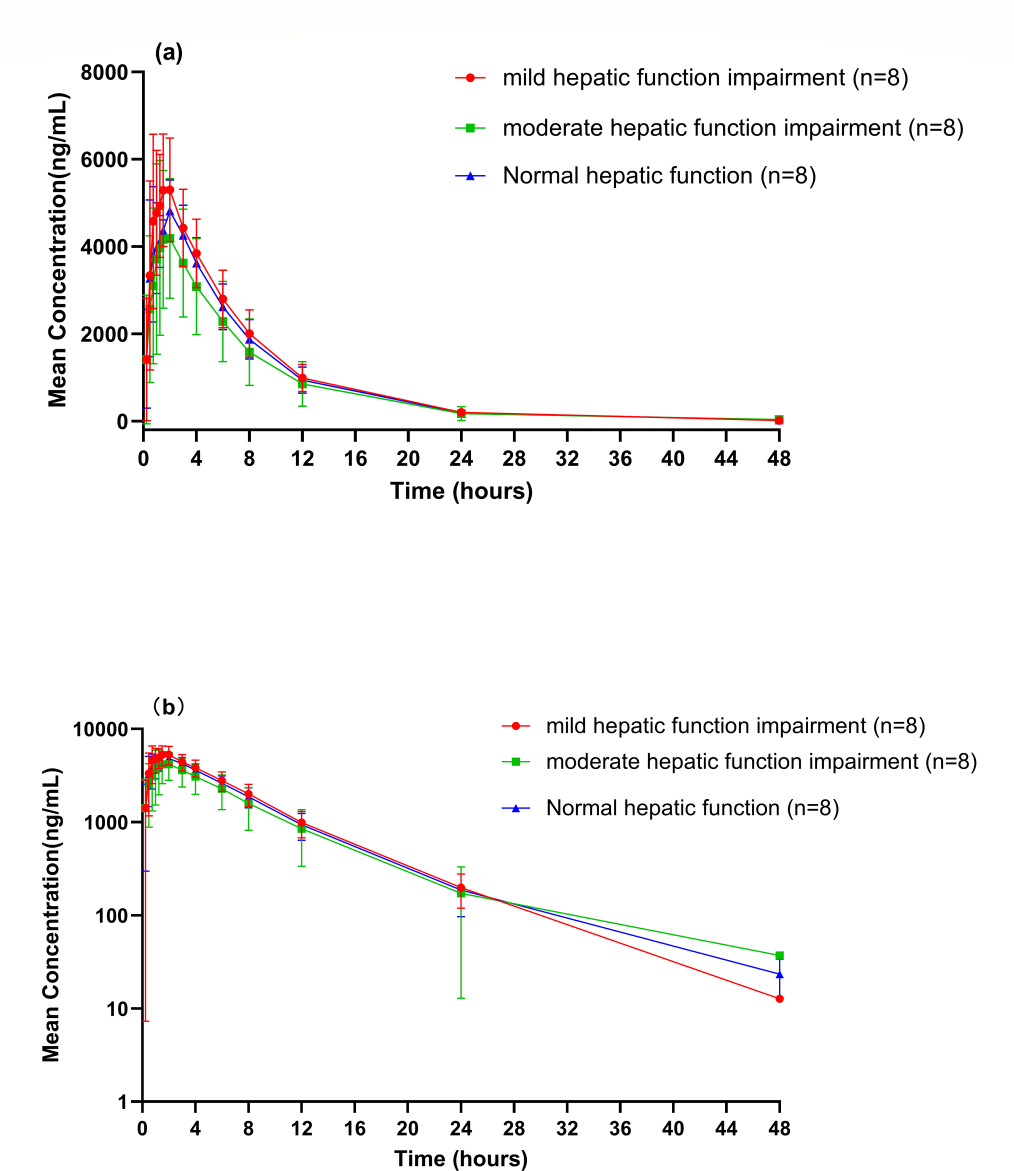
Plasma concentration-time profiles of GST-HG171 in Chinese subjects with normal or impaired hepatic function: (**a**) linear plot and (**b**) semi-log plot.

After a single oral administration of 150 mg GST-HG171, the average plasma concentration-time curve and semi-logarithmic curve of GST-HG171 in subjects with mild liver impairment, moderate liver impairment, and normal liver function are shown in Fig. 1. The main PK parameters are detailed in Table 2, and the comparative analysis of GST-HG171 PK parameters between subjects with liver dysfunction and those with normal liver function is presented in Table 3. The results of the variance analysis model showed that compared to subjects with normal liver function, the geometric mean ratio (mild/normal) and its 90% confidence interval of *C*_max_, AUC_0-*t*_, and AUC_0-∞_ for GST-HG171 in subjects with mild liver impairment were 1.14 (0.99, 1.31), 1.07 (0.88, 1.30), and 1.07 (0.88, 1.29), respectively (Table 3). For subjects with moderate liver impairment compared to those with normal liver function, the geometric mean ratio (moderate/normal) and its 90% confidence interval of *C*_max_, AUC_0-*t*_, and AUC_0-∞_ were 0.87 (0.70, 1.07), 0.82 (0.61, 1.10), and 0.82 (0.61, 1.10), respectively (Table 3).

**TABLE 2:** Pharmacokinetic parameters of GST-HG171^*a*^

PK parameter	Mild hepatic impairment (*N* = 8)	Moderate hepatic impairment (*N* = 8)	Normal hepatic function (*N* = 8)
*C*_max_ (ng/mL)	5,996.92 ± 1,171.03	4,687.04 ± 1,641.65	5,184.30 ± 460.18
AUC_0-*t*_ (h ng/mL)	42,412.87 ± 10,102.05	34,513.47 ± 16,390.60	39,530.79 ± 8,673.14
AUC_0-∞_ (h ng/mL)	43,252.82 ± 9,925.10	35,281.28 ± 16,500.92	40,251.65 ± 8,391.03
*T*_1/2_ (h)	4.66 ± 0.59	4.60 ± 0.94	4.83 ± 0.84
*T*_max_ (h)	1.25 (0.75, 2.00)	1.37 (0.75, 2.00)	1.50 (0.50, 2.00)
CLz (mL/h)	3,634.77 ± 840.22	4,979.67 ± 1,973.87	3,851.49 ± 688.39
Vz (mL)	24,006.21 ± 4,275.39	30,833.45 ± 6,882.99	26,227.82 ± 2,398.91

^*a*^ Values are mean ± standard deviation or median (minimum, maximum). *C*_max_, maximum plasma concentration; AUC_0-*t*_, area under the concentration-time curve up to the last quantifiable time; AUC_0-∞_, area under the plasma concentration-time curve from time 0 extrapolated to infinity; *T*_1/2_, terminal elimination half-life; *T*_max_, time to reach maximum plasma concentration; CL/F, apparent total body clearance; Vz/F, apparent volume of distribution based on the terminal phase.

**TABLE 3:** Ratios for pharmacokinetic parameters of GST-HG171in subjects with hepatic impairment versus normal hepatic function^*a*^

Pharmacokinetic parameters	Ratio (hepatic impairment/normal)	90% CI (%)
Mild hepatic impairment		
*C*_max_ (ng/mL)	1.14	0.99, 1.31
AUC_0-*t*_ (h ng/mL)	1.07	0.88, 1.30
AUC_0-∞_ (h ng/mL)	1.07	0.88, 1.29
Moderate hepatic impairment		
*C*_max_ (ng/mL)	0.87	0.70, 1.07
AUC_0-*t*_ (h ng/mL)	0.82	0.61, 1.10
AUC_0-∞_ (h ng/mL)	0.82	0.61, 1.10

^*a*^ CI, confidence interval.

### Safety and tolerability

A single dose of 150 mg GST-HG171 demonstrated good safety and tolerability in patients with mild and moderate liver impairment. Safety assessment was conducted for all 24 subjects enrolled in this study. There were no deaths or serious adverse events reported, and no adverse events of Grade III or above related to the study drug or leading to discontinuation occurred. Drug-related TEAEs and the incidences are summarized in Table 4. Among all TEAEs related to the drug, only one case of hypokalemia required medical intervention, while most resolved spontaneously without intervention. Based on safety assessments, there were no meaningful trends observed in important indicators such as ALT, AST, creatinine, uric acid, low-density lipoprotein (LDL) cholesterol, triglycerides, urinalysis, white blood cells, neutrophils, PR interval, blood pressure, and heart rate before and after drug administration. Evaluation of QTc interval changes revealed no prolongation (>60 ms from baseline) or values beyond the normal range. Only one subject with moderate liver impairment experienced a Grade III adverse event (decreased platelet count), with a baseline value at Grade II, while all other TEAEs were of Grade I or II severity. Among the 24 enrolled subjects in this study, a total of 8 subjects (33.3%) experienced 12 TEAEs related to the investigational drug. The incidence rates of TEAEs in the mild, moderate, and normal liver function groups were 37.5% (3/8), 62.5% (5/8), and 0% (0/8), respectively. As the degree of liver impairment increased, the incidence of adverse reactions also increased. In subjects with moderate liver impairment, adverse reactions such as decreased platelet count (50%) and hypoalbuminemia (25%) had relatively higher occurrence rates.

**TABLE 4:** Drug-related TEAEs^*a*^

	Mild hepatic impairment (*N* = 8)	Moderate hepatic impairment (*N* = 8)	Normal hepatic function (*N* = 8)
Thrombocytopenia	25% (2/8）	50% (4/8）	0
Hypoalbuminemia	0	25% (2/8）	0
Elevated alanine aminotransferase levels	0	12.5% (1/8)	0
Diarrhea	12.5% (1/8)	12.5% (1/8)	0
Hypokalemia	12.5% (1/8)	0	0

^*a*^ The data are presented as *n* (%).

## DISCUSSION

GST-HG171 is a potent, broad-spectrum, orally bioavailable small-molecule 3C-like protease inhibitor that was recently approved for treating mild to moderate COVID-19 patients in China. In preclinical studies, GST-HG171 demonstrated greater potency and efficacy compared to nirmatrelvir *in vitro* and *in vivo*. It also has good oral bioavailability and favorable safety profiles in preclinical and clinical studies. In Phase I clinical studies in healthy individuals, it demonstrated good safety and PK profiles with exposure levels in human plasma four times higher than Paxlovid within the same dose range (18). In a randomized, double-blind, placebo-controlled Phase II/III trial, GST-HG171 demonstrated both shortened median time to sustained recovery of clinical symptoms compared to placebo and negative conversion of SARS-CoV-2 nucleic acid versus placebo, with the LS mean difference in viral load change from baseline reaching the largest at day 5 of 1.75log_10_ copies/mL (20).

Results from preclinical metabolism studies *in vitro* indicated that CYP3A4 is the primary metabolic enzyme for GST-HG171 with the liver being the major organ of metabolism. Although metabolic conversion studies and evaluation of metabolites were not conducted in preliminary trials, and it cannot be definitively stated whether liver metabolism and/or excretion account for a significant portion of the absorbed drug, concomitant administration of GST-HG171 with ritonavir can inhibit CYP3A4 activity and decrease clearance, resulting in 3–6-fold increased exposure to GST-HG171 (18). Therefore, in consideration of FDA guidelines, we conducted the pharmacokinetic and safety study of GST-HG171 in subjects with liver impairment.

This study evaluated the impact of mild and moderate liver impairment on plasma exposure of GST-HG171 as well as the safety and tolerability of GST-HG171 in individuals with mild and moderate liver impairment. The results showed that compared to healthy subjects with normal liver function, there were no significant changes in the peak time (*T*_max_) and elimination half-life (*T*_1/2_) of GST-HG171 in patients with liver impairment. In subjects with mild liver impairment compared to those with normal liver function, the *C*_max_ of GST-HG171 increased by approximately 14%, and AUC_0-*t*_ and AUC_0-∞_ increased by approximately 7%. In subjects with moderate liver impairment compared to those with normal liver function, the *C*_max_ of GST-HG171 decreased by approximately 14%, and AUC_0-*t*_ and AUC_0-∞_ decreased by approximately 18%. These exposure changes were clinically insignificant, indicating that liver impairment has minimal effect on the exposure to GST-HG171. Therefore, it is anticipated that a dose adjustment is not necessary for subjects with mild and moderate liver impairment. However, it should be noted that the approved treatment regimen for GST-HG171 is 150 mg of GST-HG171 combined with 100 mg of ritonavir, administered twice daily for 5 consecutive days. This study, however, involved a single-dose administration of GST-HG171 in subjects with mild to moderate hepatic impairment. Therefore, in post-marketing multiple-dose treatments of COVID-19 patients with mild to moderate hepatic impairment, the incidence and severity of adverse reactions may increase.

Among the 24 subjects enrolled in this study, 8 subjects (33.3%) reported 12 TEAEs related to the investigational drug. There were no occurrences of serious adverse events, serious adverse reactions, TEAEs leading to discontinuation, or TEAEs leading to withdrawal. Single-dose administration of GST-HG171 was well tolerated in subjects with normal liver function and mild to moderate liver impairment. No adverse reactions related to taste perversion, which is common with nirmatrelvir/ritonavir, were reported in this study (6). However, in subjects with moderate liver impairment, adverse reactions such as decreased platelet count (50%) and hypoalbuminemia (25%) had relatively higher occurrence rates, which may be related to the baseline thrombocytopenia and lower albumin levels in patients with moderate liver impairment. Nonetheless, adverse reactions of platelet count decrease and hypoalbuminemia were not observed in the normal liver function group. Despite the minimal impact of liver impairment on the exposure to GST-HG171, increased sensitivity to the investigational drug in liver-damaged populations may lead to decreases in platelets and albumin. Therefore, it is advisable to continue monitoring changes in platelet count and albumin levels in patients with moderate liver impairment. The study’s limitations include a small sample size and the exclusion of PK and safety evaluations in patients with severe hepatic impairment. Severe liver impairment was not assessed due to expected significant increases in GST-HG171’s AUC, posing high risk. Similar drugs, such as Paxlovid, are contraindicated in severe hepatic impairment. Another limitation is the gender imbalance among the study participants with relatively few female subjects in each group. Therefore, the conclusions of this study are not fully applicable to female patients with hepatic impairment.

### Conclusion

The pharmacokinetic results of GST-HG171 in Chinese subjects with mild and moderate liver impairment and those with normal liver function in this study demonstrated the minimal impact of mild to moderate liver impairment on the exposure to GST-HG171, suggesting that patients with mild to moderate liver impairment do not require a dose adjustment of GST-HG171 tablets in the clinic. GST-HG171 has good safety and tolerability after single-dose administration in subjects with normal liver function as well as those with mild to moderate liver impairment.
